# Validation of the 8th lung cancer TNM classification and clinical staging system in a German cohort of surgically resected patients

**DOI:** 10.1515/iss-2020-0010

**Published:** 2020-08-12

**Authors:** Samantha Taber, Joachim Pfannschmidt

**Affiliations:** Department of Thoracic Surgery, Heckeshorn Lung Clinic–HELIOS Klinikum Emil von Behring, Berlin, Germany

**Keywords:** lung cancer, NSCLC, prognosis, staging, thoracic surgery, TNM, validation

## Abstract

**Objectives:**

The updated 8th edition of the tumor, node, metastases (TNM) classification system for non-small cell lung cancer (NSCLC) attempts to improve on the previous 7th edition in predicting outcomes and guiding management decisions. This study sought to determine whether the 8th edition was more accurate in predicting long-term survival in a European population of surgically treated NSCLC patients.

**Methods:**

We scanned the archives of the Heckeshorn Lung Clinic for patients with preoperative clinical stages of IIIA or lower (based on the 7th edition), who received surgery for NSCLC between 2009 and 2014. We used pathologists’ reports and data on tumor size and location to reassign tumor stages according to the 8th edition. We then analyzed stage specific survival and compared the accuracy of the two systems in predicting long-term survival. We excluded patients with neoadjuvant treatment, incomplete follow-up data, tumor histologies other than NSCLC, or death within 30 days of surgery.

**Results:**

The final analysis included 1,013 patients. Overall five-year survival was 47.3%. The median overall survival (OS) was 63 months (range 1–222), and the median disease-free survival (DFS) was 50 months (0–122). The median follow-up time for non-censored patients was 84 months (range 60–122).

**Conclusions:**

We found significant survival differences between the newly defined stages 1A1, 1A2 and 1A3 (previously 1A). We also found that the 8th edition of TMN classification was a significantly better predictor of long-term survival, compared to the 7th edition.

## Introduction

The tumor, node, and metastasis (TNM) classification scheme for non-small cell lung cancer (NSCLC) aims to provide a standardized means of describing the anatomic extent of the cancer at time of diagnosis. It is important for individual patients and clinicians in determining prognosis and appropriate course of treatment [1]. It also has a role in facilitating communication between clinicians and researchers from different disciplines and geographical locations. Although this requires a certain degree of stability in the nomenclature, the Union for International Cancer Control (UICC), which oversees the TNM classification system internationally, periodically makes updates to reflect developments in technology and understanding of tumor behavior [2].

The most recent 8th edition of the TNM classification system has been in effect since January 2017 [3]. Although the update includes modifications to both the T and M descriptors [4], for patients under consideration for surgery (nearly 85% of the database) [5], the T descriptor is of primary interest. The 8th edition changes cause a small proportion of patients to be assigned a lower T category (based on tumor atelectasis, involvement of the main bronchus, or invasion of the mediastinal pleura). For the majority of patients, however, the changes in the 8th edition result in a higher T category and consequently a higher UICC stage. Based on the idea that every centimeter of tumor size affects prognosis, the T descriptor for the smallest tumors (stage IA in the 7th edition) has been further differentiated into stages IA1 (T1mi or 1a N0M0), IA2 (T1b N0M0) and IA3 (T1c N0M0). Moreover, tumors 4 cm and greater may now be assigned a higher T category than previously [6].

The UICC recommendations are based on a series of analyses performed by the International Association for the Study of Lung Cancer (IASLC) [] and are based on an international database of nearly 80,000 patients [2]. The 8th edition was externally validated in a large study of North American patients from the National Cancer Data Base [9]. The original database, however, drew a disproportionate number of patients from Asia (specifically Japan), and most other existing validation studies for surgically treated patients, are based on Asian populations [10], [11], [12], [13]. This is significant in light of the known tumor biological and prognostic differences between Asian and Caucasian populations [14], [15]. Even within the IASLC database, early stages of NSCLC seem to predominate within Asian populations, while advanced stages are more prevalent in European populations [5]. Validation studies of the 8th edition of the TNM classification scheme in European populations are limited [16], [17]. The object of this study is to determine whether the 8th edition is more accurate than the 7th edition in predicting long-term outcome in a Germany-based population of surgically treated NSCLC patients.

## Methods

We retrospectively scanned the archives of the Heckeshorn Lung Clinic in Berlin, Germany for patients who had undergone lung resection for NSCLC between January 2009 and June 2014. Data on these patients had been collected for purposes of internal quality control, and all patients had given their informed written consent for their data to be used in future research projects. For this reason the institutional review board waived the requirement for registration.

All patients included had preoperative clinical stages of IIIA (T1a-T2b N2M0 or T3-T4 N1M0 or T4 N0M0) or lower (based on the 7th edition, with some cases of postoperative pathology-based upstaging to stage IIIB). We excluded patients with neoadjuvant treatment, death within 30 days of surgery, tumor histology other than NSCLC, or incomplete follow-up data.

Preoperative evaluation included detailed medical history, physical examination, positron emission tomography CT (PET-CT), and pulmonary function tests. Cranial CT or cranial MRT were only performed when symptoms suggestive of cerebral metastases were present. All patients received surgery in curative intent. Based on local tumor board consensus, selected patients also received adjuvant chemotherapy, radiation therapy, or combined radiochemotherapy.

All resected tissues, including lymph nodes, were examined by a board-certified pathologist and assigned a tumor stage based on the 7th edition TNM classification for NSCLC. We retrospectively extracted the data on tumor size and location from pathologists’ reports and reassigned each patient new TNM and UICC stages based on the 8th edition guidelines.

Overall survival time (OS) was defined as date of surgery until either the date of death (from any cause) or the date when the patient was last known to be alive. Disease free survival (DFS) was defined as date of surgery until date of tumor recurrence. Patients were followed-up with physical examination and chest CT, first biannually and after two years, annually. In-hospital follow-up data was supplemented with reports from external physicians and information from the local residents’ registration office. Patients with no known date of death, for whom data was not available for at least five years post-surgery, were considered lost to follow-up and were excluded from the analysis. Patients known to be alive at the end of the five-year follow-up period were censored. In cases where patients received a second surgery for recurrent lung cancer, only the date of the initial surgery was included in the analysis for determining OS and DFS.

Finally, we retrospectively extracted additional patient data on age, gender, tumor histology, extent of surgical resection, tumor resection margins, and anatomic location.

### Statistical analysis

All statistical analyses were performed using SPSS statistics for Windows, V.20 (IBM Corp). After reclassifying tumor stage according to the 8th edition of the TNM system, we generated Kaplan-Meier survival curves for each tumor stage, based on both the 7th and the 8th edition classification systems. We used log-rank tests determine whether the observed differences in survival curves were significant. We also evaluated potentially confounding factors (age, sex, tumor histology, extent of surgery, tumor resection margins) for significance using Chi-squared and Mann-Whitney tests and included those where p<0.05 in the Cox regression analysis. After determining the significant independent variables, we performed additional analyses of the differences in neighboring survival curves to adjust for these covariates. Finally, we determined the R^2^ measure, as recommended by the IASLC, as a means of assessing the discriminative ability of the respective models [18].

## Results

**Figure 1:**
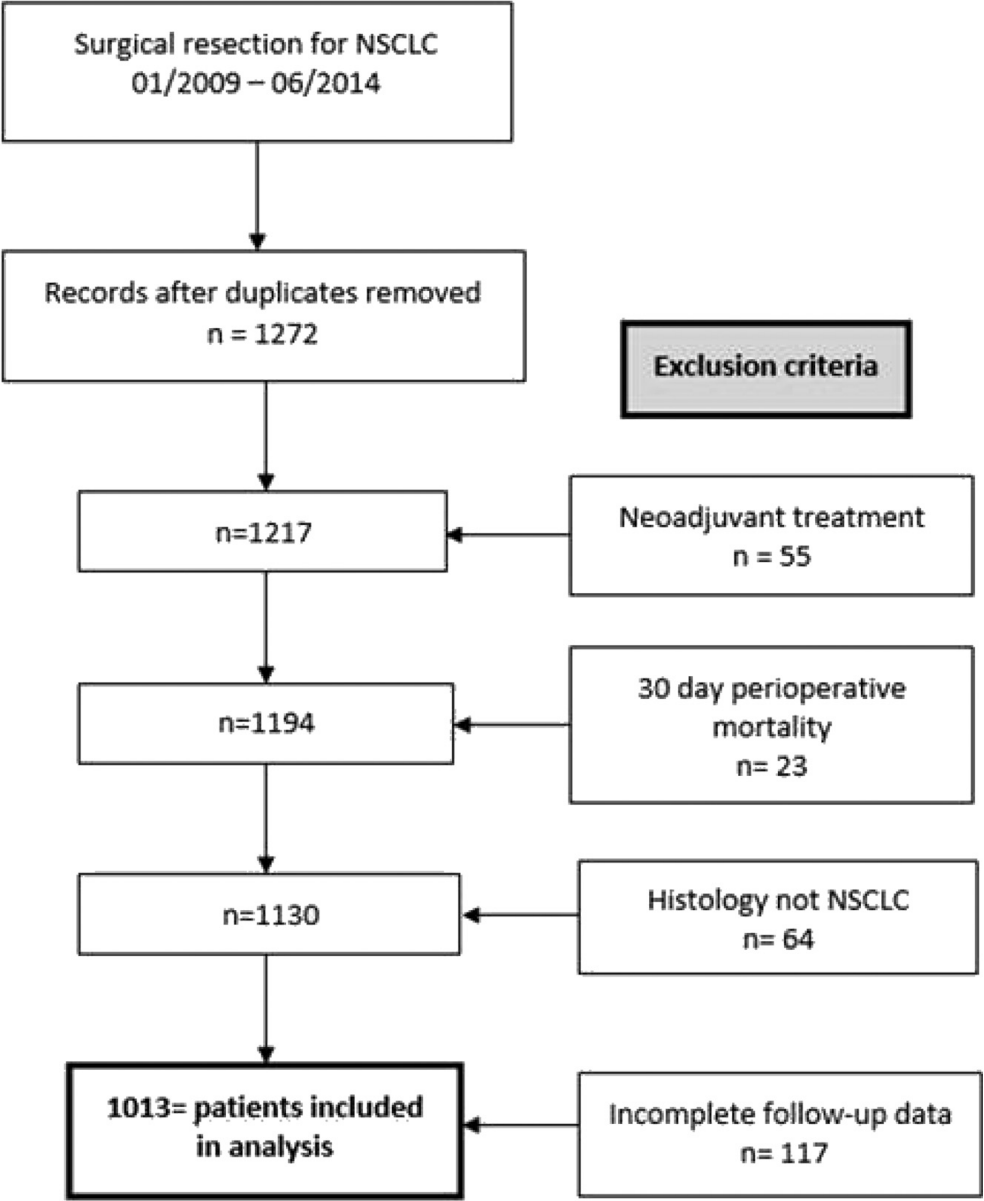
Flow-chart demonstrating patient selection; NSCLC = non-small cell lung cancer.

We initially identified 1,272 patients, who were operated on in curative intent for expected NSCLC. 55 patients were excluded for neoadjuvant treatment, and 23 were excluded for perioperative mortality (death within 30 days of surgery). 64 patients were excluded after the lesion in question turned out to be an entity other than NSCLC, and 117 were excluded for incomplete follow-up. 1,013 patients could be included in the final analysis, as is illustrated visually in Figure 1.

The baseline characteristics of the cohort are summarized in Table 1. 479 patients (47.3%) were censored (alive at the end up of a minimum follow-up period of 60 months). Among censored patients the median follow-up time was 84 months (range 60–122). For the cohort as a whole, median OS was 63 months (range 1–122), and median DFS was 50 months (range 0–122). The median age was 67 years (range 39–87), and males accounted for 58.4% of the study population (n=592). Adenocarcinoma appeared most frequently (51.7%), followed by squamous cell carcinoma (35.0%). Lobectomy or bilobectomy was the most frequently performed surgical procedure (79.2%). Pneumonectomies accounted for 7.8% of cases, while anatomical segmental resections (7.7%) and atypical wedge resections (5.5%) accounted for the rest. The median tumor diameter was 3.5 cm (IQR 2.0–5.4 cm). In most cases a tumor-free resection margin was possible (94.7%). 297 patients (29.3%) received some form of adjuvant treatment (chemotherapy: n=182, radiation: n=29, combined radiochemotherapy: n=86).

**Table 1:** Baseline characteristics of study population.

Variable	Value
*Gender*	
Male	592 (58.4%)
Female	421 (41.6%)
*Histology*	
Adeno	524 (51.7%)
Squamous	355 (35.0%)
Large cell	86 (8.5%)
Other (NSCLC)	48 (4.7%)
*Age (years)*	
≤65	443 (43.7%)
>65	570 (56.3%)
*Age (years)*	
Median (range)	67 (39–87)
*Operation*	
(Bi-)lobectomy	802 (79.2%)
Pneumonectomy	79 (7.8%)
Segmentectomy	78 (7.7%)
Wedge resection	54 (5.3%)
*Resection margins*	
R0	959 (94.7%)
R1, R2, RX	54 (5.3%)
*Side*	
Left	430 (42.5%)
Right	583 (57.6%)
*OS (months*)	
Median (IQR)	63 (1–122)
*DFS (months)*	
Median (IQR)	50 (0–122)
*Tumor diameter (cm)*	
Median (IQR)	3.5 (2.0–5.4)
*Censored (OS)*	479 (47.3%)
*Censored (DFS)*	368 (36.3%)
*Follow up censored (months)*	
Median (range)	84 (60–122)

NSCLC = non-small cell lung cancer, IQR = interquartile range, OS = overall survival, DFS = disease free survival.

Tumor stage distribution within the cohort, based on the 7th and 8th TNM editions respectively, is presented in Table 2. Of the 294 patients in stage IA (T1a or T1b N0M0) in the 7th edition, only 34 (3.4%) qualified for stage IA1 (T1a N0M0) in the 8th edition. The remaining patients in stage IA (7th edition) were reassigned to either stages IA2 (T1b N0M0) or IA3 (T1c N0M0) in the 8th edition. Further reassigning of stages resulted in a net shift of patients to higher tumor stages, as is presented in Table 2. A total of 341 patients (33.7%) were shifted to a higher tumor stage (due to tumor size). Only 11 patients (1.1%) were downstaged due to distance from carina or mediastinal pleural invasion.

**Table 2:** Changes in distribution based on 7th and 8th editions of TNM classification.

UICC stage based on 8th TNM	UICC stage based on 7th TNM						
	IA	IB	IIA	IIB	IIIA	IIIB	Total (%)
IA1 (T1aN0M0)	34	0	0	0	0	0	34 (3.36%)
IA2 (T1bN0M0)	155	0	0	0	0	0	155 (15.3%)
IA3 (T1cN0M0)	105	0	0	0	0	0	105 (10.4%)
IB (T2aN0M0)	0	112	0	0	0	0	112 (11.1%)
IIA (T2bN0M0)	0	67	24	0	0	0	91 (8.98%)
IIB (T1a-T2bN1M0, T3N0M0)	0	0	121	51	0	0	172 (17.0%)
IIIA (T1a-T2bN2M0, T3-4N1 or T4N0M0)	0	0	0	83	167	0	250 (24.7%)
IIIB (T1a-T2bN3M0, T3-4N2M0)	0	0	0	0	70	24	94 (9.3%)
Total (%)	294 (29.0%)	179 (17.7%)	145 (14.3%)	134 (13.2%)	237 (23.4%)	24 (2.37%)	

UICC = Union for International Cancer Control.

The results of the univariate analysis with respective p-values appear in Table 3. Here we determined that male gender, surgery other than lobectomy/bilobectomy, positive tumor resection margins, age >65 years, and higher tumor stage (both 7th and 8th editions) were associated with worse five-year OS and DFS. Tumor histology and side (left vs. right) were not significant. In the multivariate Cox regression analysis we found that male gender, age >65 years, segmentectomy/wedge resection vs. lobectomy, and increasing tumor stage were significant independent predictors of both worse OS and DFS. Increasing tumor stage, for both the 7th and 8th TNM editions, was associated with worse OS and DFS. These results are summarized in Table 4. 7th edition had an R^2^ of 0.142; the model based on the 8th edition had an R^2^ of 0.153. For DFS, the R^2^ values for the models based on the 7th and 8th editions were 0.128 and 0.135 respectively, suggesting that the 8^th^ edition makes for a marginally better predictive model.

**Table 3:** Univariate analysis of potential outcome factors.

Variable	five-year OS		five-year DFS	
	%	p-Value	%	p-Value
*Side*		0.144		0.456
Left	50.0		37.7	
Right	45.4		35.4	
*Gender*		<0.001		<0.001
Male	41.6		31.4	
Female	55.3		43.2	
*Histology*		0.295		0.421
Adeno	47.9		37.0	
Squamous	47.0		34.9	
Large cell	39.5		32.6	
Other NSC	56.3		45.8	
*Surgery*		<0.001		<0.001
(Bi-)lobectomy	51.1		39.5	
Pneumonectomy	43.0		35.4	
Segment	26.9		19.2	
Wedge	25.9		14.8	
*Resection margins*		0.005		0.008
R0	48.5		37.4	
R1, R2, RX	26.4		9.0	
*UICC 7*		<0.001		<0.001
IA	57.1		43.2	
IB	54.2		43.0	
IIA	55.2		40.7	
IIB	45.5		32.8	
IIIA	30.0		24.9	
IIIB	8.3		8.3	
*UICC 8*		p<0.001		p<0.001
IA1	73.5		61.8	
IA2	54.8		40.7	
IA3	55.2		41.0	
IB	57.1		50.0	
IIA	52.8		40.7	
IIB	52.3		39.0	
IIIA	36.4		28.0	
IIIB	19.2		18.1	

OS = overall survival; DFS = disease free survival; UICC = Union for International Cancer Control.

**Table 4:** Results from the Cox regression analysis for OS and DFS according to 7th and 8th TNM classifications.

OS based on 7th TNM				DFS based on 7th TNM			
Variable	p-Value	HR	95% CI	Variable	p-Value	HR	95% CI
*Gender*				*Gender*			
Male	Reference			Male	Reference		
Female	<0.001	0.79	0.72–0.87	Female	<0.001	0.86	0.79–0.93
*Age*				*Age*			
≤65 years	Reference			≤65 years	Reference		
>65 years	<0.001	1.22	1.11–1.33	>65 years	<0.001	1.20	1.11–1.30
*Surgery*				*Surgery*			
(Bi-)lobectomy	Reference			(Bi-)lobectomy	Reference		
Pneumonectomy	<0.001	0.66	0.51–0.86	Pneumonectomy	<0.001	0.67	0.53–0.86
Segmentectomy	<0.001	1.30	1.01–1.58	Segmentectomy	0.116	1.19	0.96–1.48
Wedge resection	<0.001	1.52	1.17–1.98	Wedge resection	<0.001	1.58	1.24–2.02
*UICC 7*				*UICC 7*			
Stage IA	Reference			Stage IA	Reference		
Stage IB	<0.001	0.68	0.55–0.83	Stage IB	<0.001	0.69	0.57–0.84
Stage IIA	<0.001	0.67	0.53–0.84	Stage IIA	<0.001	0.68	0.56–0.83
Stage IIB	0.29	0.89	0.72–1.10	Stage IIB	0.628	0.95	0.78–1.16
Stage IIIA	<0.001	1.62	1.38–1.92	Stage IIIA	<0.001	1.53	1.31–1.79
Stage IIIB	<0.001	2.53	1.75–3.67	Stage IIIB	<0.001	2.20	1.52–3.16
**OS based on 8th TNM**				**DFS based on 8th TNM**			
*Gender*				*Gender*			
Male	Reference			Male	Reference		
Female	<0.001	0.80	0.73–0.88	Female	<0.001	0.86	0.79–0.93
*Age*				*Age*			
≤65 years	Reference			≤65 years	Reference		
>65 years	<0.001	1.21	1.11–1.33	>65 years	<0.001	1.19	1.1–1.3
*Surgery*				*Surgery*			
(Bi-)lobectomy	Reference			(Bi-)lobectomy	Reference		
Pneumonectomy	<0.001	0.62	0.48–0.80	Pneumonectomy	<0.001	0.63	0.49–0.81
Segmentectomy	0.013	1.34	1.06–1.69	Segmentectomy	0.04	1.26	1.01–1.57
Wedge resection	<0.001	1.63	1.25–2.11	Wedge resection	<0.001	1.65	1.30–2.10
*UICC 8*				*UICC 8*			
Stage IA1	Reference			Stage IA1	Reference		
Stage IA2	0.08	0.81	0.64–1.03	Stage IA2	0.04	0.81	0.66– 0.99
Stage IA3	0.21	0.84	0.64–1.11	Stage IA3	0.31	0.88	0.70–1.12
Stage IB	0.07	0.78	0.59–1.02	Stage IB	0.04	0.78	0.61–0.99
Stage IIA	0.95	0.99	0.75–1.32	Stage IIA	0.68	0.95	0.74–1.22
Stage IIB	0.29	0.89	0.71–1.11	Stage IIB	0.20	0.88	0.72–1.07
Stage IIIA	<0.001	1.66	1.39–1.99	Stage IIIA	<0.001	1.53	1.30–1.80
Stage IIIB	<0.001	3.05	2.41–3.87	Stage IIIB	<0.001	2.59	2.06–3.26

OS = overall survival, DFS = disease free survival, CI = confidence interval; HR = hazard ratio; UICC = Union for Inter-national Cancer Control.

**Figure 2:**
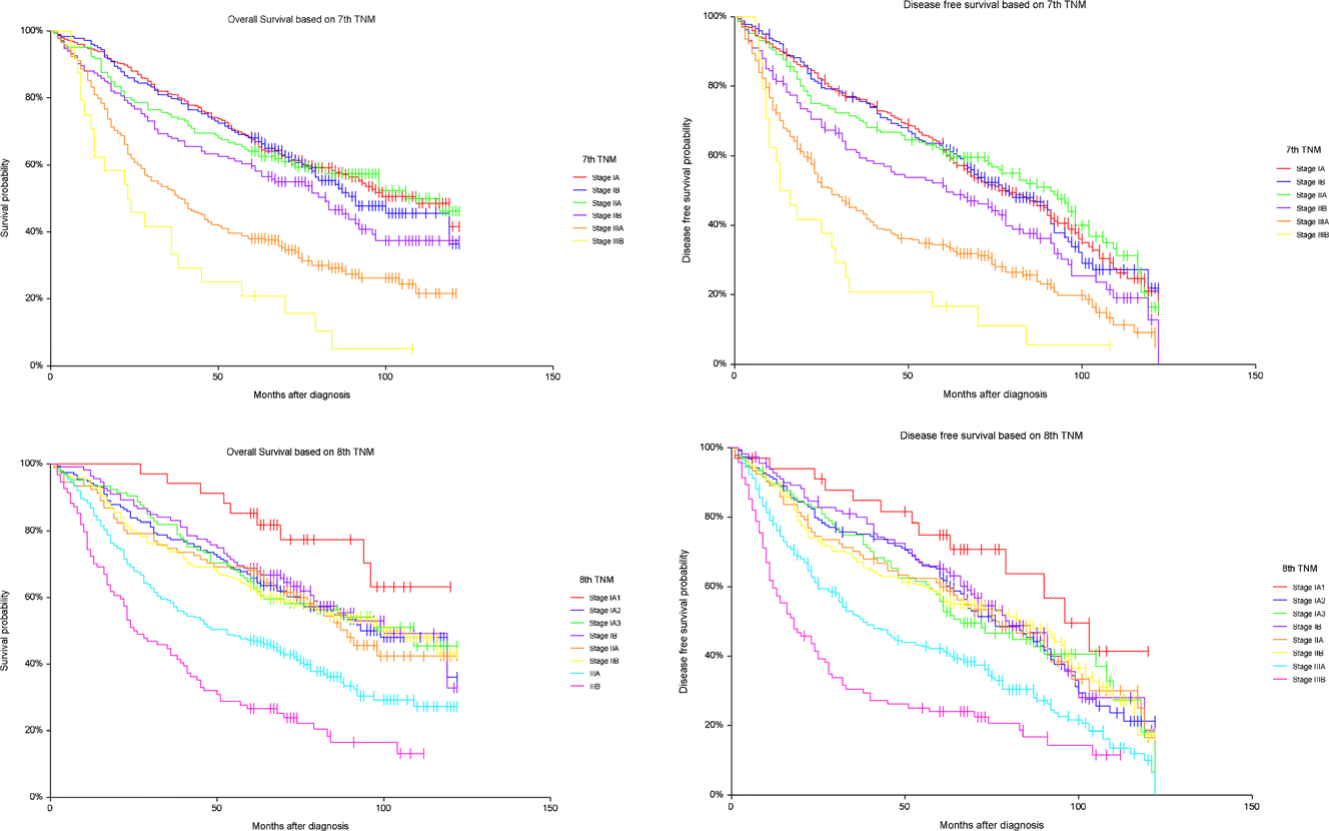
Kaplan-Meier curves with overall survival (OS) and disease free survival (DFS) by stage based on the 7th and 8th editions of TNM respectively. Log rank analysis of OS based on 7th TNM: IA vs. IB (p=0.6165), IB vs. IIA (p=0.812), IIA vs. IIB (p=0.085), IIB vs. IIIA (p<0.001), IIIA vs. IIIB (p=0.017); OS based on 8th TNM: IA1 vs 1A2 (p=0.039), IA2 vs. IA3 (p=0.998), IA3 vs. IB (p=0.681), IB vs. IIA (p=0.494), IIA vs. IIB (p=0.849), IIB vs. IIIA (p=<0.001), IIIA vs. IIIB (p<0.001); DFS based on 7th TNM: IA vs. IB (p=0.946), IB vs. IIA (p=0.630), IIA vs. IIB (p=0.019), IIB vs. IIIA (p=0.003), IIIA vs. IIIB (0.043); DFS based on 8th TNM: IA1 vs. 1A2 (p=0.084), IA2 vs. IA3 (p=0.775), IA3 vs. IB (p=0.484), IB vs. IIA (p=0.600), IIA vs. IIB (p=0.997), IIB vs. IIIA (p<0.001), IIIA vs. IIIB (p<0.001).

Figure 2 shows the survival curves for OS and DFS according to UICC tumor stage for both the 7th and 8th TNM editions respectively. We observed stepwise deterioration with increasing pathologic stage. In the initial log-rank analysis, not all differences in adjacent survival curves were significant (Figure 2), but after adjusting for the significant predictors identified in the initial Cox regression analysis (age, surgery, and gender), the differences between all neighboring stages were significant, as is presented in Table 5.

**Table 5:** Comparison of neighboring Union for International Cancer Control (UICC) stages: Cox regression analysis after adjusting for gender, surgery, and age.

OS based on 7th edition TNM			
Stage	p-Value	HR	95% CI
IA vs. IB	<0.001	0.61	0.51–0.73
IB vs. IIA	<0.001	0.41	0.31–0.54
IIA vs. IIB	<0.001	0.27	0.20–0.38
IIB vs. IIIA	<0.001	0.24	0.17–0.34
IIIA vs. IIIB	<0.001	0.39	0.27–0.57
**OS based on 8th edition TNM**			
IA1 vs. 1A2	0.004	0.42	0.24–0.75
IA2 vs. IA3	<0.001	0.34	0.20–0.59
IA3 vs. IB	<0.001	0.29	0.17–0.48
IB vs. IIA	<0.001	0.22	0.14–0.36
IIA vs. IIB	<0.001	0.22	0.15–0.33
IIB vs. IIIA	<0.001	0.20	0.14–0.27
IIIA vs. IIIB	<0.001	0.33	0.26–0.42
**DFS based on 7th edition TNM**			
IA vs. IB	<0.001	0.66	0.56–0.78
IB vs. IIA	<0.001	0.46	0.36–0.59
IIA vs. IIB	<0.001	0.31	0.23–0.42
IIB vs. IIIA	<0.001	0.30	0.21–0.42
IIIA vs. IIIB	<0.001	0.46	0.32–0.66
**DFS based on 8th edition TNM**			
IA1 vs. 1A2	0.015	0.55	0.34–0.89
IA2 vs. IA3	<0.001	0.44	0.28–0.70
IA3 vs. IB	<0.001	0.39	0.25–0.61
IB vs. IIA	<0.001	0.30	0.20–0.46
IIA vs. IIB	<0.001	0.29	0.20–0.41
IIB vs. IIIA	<0.001	0.25	0.19–0.34
IIIA vs. IIIB	<0.001	0.39	0.31–0.48

OS = overall survival; DFS = disease free survival; HR = hazard ratio; CI = confidence interval.

## Discussion

In contrast to many tumor classification systems, the TNM staging system for NSCLC is not based on consensus and expert opinion but on an extensive international database and a series of complex statistical analyses. Despite these efforts, the IASLC’s most recent 8th edition draws a disproportionate amount of data from Asia and from Japan specifically [18], raising questions about the applicability to non-Asian populations. Moreover, most existing validation studies are based on Asian populations. This study attempts to determine how well the 8th edition of the TNM classification system predicts long-term outcome in a European population of surgically treated NSCLC patients.

The primary finding of this study is that the revised 8th edition TNM is more accurate at predicting long-term OS and DFS than the previous 7th edition. The improvements, however, are small and may not apply equally to all tumor stages. The integrated predictive models that included both tumor stage and covariates were slightly better at predicting OS and DFS (based on R^2^ values) when incorporating 8th edition stages rather than 7th edition stages. Sui et al. also found that the 8th edition made for somewhat better predictive models (R^2^=0.172 vs. 0.162 for OS; R^2^=0.183 vs. 0.178 for DFS) [11], and most other validation studies came to similar conclusions [11], [12], [13], [19]. Only Jung et al., analyzing the T descriptor changes in a Korean population (n=1,316), did not find any difference [10]. Interestingly, the findings of the other European studies we identified were less conclusive. Blaauwgeers et al. (n=683, Netherland) only included patents with pT3 pN0 tumors and found that the 8th edition was only a better predictor for certain tumor constellations [17]. Neppl et al. (n=354, Switzerland) found that the 8^th^ edition was slightly better at predicting long-term outcome but only in patients with squamous cell carcinomas [16].

After adjusting for covariates, all neighboring tumor stages showed significant deterioration in both OS and DFS with increasing tumor stage (Table 5). Of our observed survival curves, the outcome differences between the newly created 8th edition stages IA1 and IA2 is perhaps most noteworthy (hazard ratio: IA1 vs. IA2 = 0.42 for OS; hazard ratio: IA1 vs. IA2 = 0.55 for DFS). Even before adjusting for covariates, in comparing stages IA1 and IA2 we observed a significant difference for OS (p=0.039) and a trend for DFS (p=0.08), supporting the notion that even in very early stages small size differences can matter. Chen et al. found that the survival curve differences were only significant for stages IA1 vs IA2 and for stages IA2 vs IA3 [12]. In our study the prognostic differences between stages IA2 through IIA (T2b N0M0) were less pronounced, but we observed a clear drop in survival rates (OS and DFS) going from stage IIB (T1a-T2b N1M0 or T3 N0M0) to IIIA (T1a-T2b N2M0 or T3-2 N1M0 or T4 N0M0), and from stage IIIA to IIIB (T1a-T3 N3M0 or T4 N2) (Figure 2).

In initial discussions and validation analyses the 7th edition was criticized for having too few patients in stage IIB [20], [21]. The 8th edition changes regarding tumor size result in the upstaging of tumors previously classified as stage IIA to stage IIB. In our study the proportion of patients in stage IIB grew from 13 to 16%, while the proportion in stage IIA (Tumor 4–5 cm, no affected lymph nodes) decreased from 14 to 9%. Sui et al. reported that after reclassifying according to the 8th edition, the proportion of stage IIA tumors decreased to 5.5% [7], [11].

Perhaps more importantly, the revisions to the 8th edition mean that patients with tumors greater than 4 cm (≤5 cm, no nodal metastases), are upstaged from stage IB to stage IIA. These shifts may affect decisions to recommend adjuvant chemotherapy or not. In our study this applied to 67 patients, who were retrospectively upstaged to stage IIA and may have been offered adjuvant chemotherapy if diagnosed and staged today on the basis of the 8th edition. It is unclear, however, whether this subset of patients benefits from adjuvant chemotherapy or not [22], [23], [24].

The other critical shift is from stage IIIA to IIIB. Apart from a small proportion of cases that are downstaged from T3 to T2 on the basis of tumor atelectasis, carina proximity or invasion of the mediastinal pleura, most 8th edition shifts are upward from stage IIIA to IIIB, for patients with N2 lymph nodes and tumors larger than 4 cm. Our cohort contained 70 such patients with 7th edition stage IIIA tumors that were retrospectively upstaged to stage IIIB. It is unclear whether these patients would have been offered surgery if diagnosed today, although decisions to perform surgery or not are additionally complicated by the well-established discrepancies between clinical and pathological staging [25].

With advances in genomic analysis and targeted therapies, genetic differences even among tumors of the same histological subtype are becoming more significant than ever. A tumor mutation that responds well to available immunotherapies can mean a significant survival benefit that is independent of tumor stage [26], [27]. Developments in the field of cancer immunology have sparked discussions about incorporating tumorbiological characteristics into future staging systems, but so far no actual changes have been implemented.

As secondary findings we determined that female gender and age 65 or younger were significantly independently associated with better outcomes, in concordance with other studies [28], [29]. Additionally, pneumonectomy was a significant independent predictor of better OS and DFS, while segmentectomy and wedge resection were significant independent predictors of worse long-term outcome. These findings, however, must be interpreted in the context of the fact that lobectomy or bilobectomy is our standard approach (79.2% in our cohort). The seeming benefit of pneumonectomy is likely an artifact due to patient selection. Not all patients who can tolerate lobectomy can tolerate pneumonectomy, meaning that only healthier patients can even be considered for pneumonectomy. Moreover, pneumonectomies are associated with significantly greater perioperative mortality, and the fact that we excluded patients who died within 30 days of surgery may also help explain the seemingly better outcomes for patients with pneumonectomies. Due to the enormous volume loss that pneumonectomy entails it cannot be recommended for peripheral tumors.

Interestingly, histology did not have a significant impact on outcome. Although patients with large cell carcinomas had worse five-year OS (39.5%), the five-year survival rates for patients with adenocarcinomas and squamous cell carcinomas were nearly the same (47.9 vs 47.0%). Although tumor free resection margins had a significant positive impact on OS and DFS in the univariate analysis, this effect was not a significant independent predictor in the multivariate analysis. This is somewhat surprising but may have to do with the small number of patients with positive resection margins (5.3%). It may also be that the effect was small enough to be outweighed by the more powerful effects of the other factors discussed above.

This study incorporates a large number of patients followed until death or for a minimum of five years following surgery. It is also one of the only broader investigations of the 8th edition of the TNM classification in a European population. However, it has some limitations. As in all retrospective studies a certain degree of bias is unavoidable. Additionally, it was not possible to incorporate data on comorbidities, which for obvious reasons can affect OS, but may potentially affect DFS as well because of decisions to forgo adjuvant treatment for example. We were able to partially account for comorbidity and non-oncological causes of death by excluding patients who died within 30 day of surgery. While not a limitation per se, it is also important to underscore that the data applies only to surgically treated patients.

Finally, although the 8th edition seems to be slightly better at predicting OS and DFS the difference is small. The differences of all neighboring survival curves were significant, but in most cases only after controlling for the identified covariates of gender, age, and surgery type. Although tumor stage influences outcome in surgical patients with NSCLC it is only one prognostic factor of many. In summary, this analysis validates the revised 8th edition stage groupings for the TNM classification for NSCLC in a large European cohort of surgical patients. The significant differences in outcomes between stages IA1 and IA2 and IA3 support the 8th edition creation of these new categories.
