# Supplementary material for: Validation of the 8th lung cancer TNM classification and clinical staging system in a German cohort of surgically resected patients
Source: Innov Surg Sci. 2020 Aug 12;5(1-2):1–9. doi: 10.1515/iss-2020-0010 (PMC7798301; doi:10.1515/iss-2020-0010)
Supplement: Supplementary file 1 [file iss-5-20200010-s001.pdf]

Reviewer Assessment

Joachim Pfannschmidt & Samantha Taber: Validation of the 8th lung cancer TNM classification and clinical staging system in a German cohort of surgically resected patients

Reviewers' Comments to Original Submission

Reviewer 1: anonymous

Date received: 03-Apr-2020
Reviewer recommendation: **Return to author for minor modifications**
Reviewer overall scoring: **High**

Assessment Form scores: 5 = High/Yes; 3 = Medium/Adequate; 1 = Low

Is the subject area appropriate for the journal					
Does the title clearly reflect the paper's content?	5				
Does the abstract clearly reflect the paper's content	5				
Do the keywords clearly reflect the paper's content?	5				
Does the introduction present the problem clearly?	5				
Are the results/ conclusions justified?	5				
How comprehensive and up-to-date is the subject matter presented?	5				
How adequate is the data presentation?	5				
Are units and terminology used correctly?	5				
Is the number of cases adequate?	5				
Are the experimental methods/ clinical studies adequate?	5				
Is the length appropriate in relation to the content?			3		
Does the reader get new insights from the article?		4			
Please rate the practical significance.		4			
Please rate the accuracy of methods.	5				
Please rate the statistical evaluation and quality control.	5				
Please rate the appropriateness of the figures and tables.	5				
Please rate the appropriateness of the references.	5				
Please evaluate the writing style and use of language.	5				
Please judge the overall scientific quality of the manuscript.		4			
Are the methods used worthy of reproduction in greater deal?	yes				

Comments to author: Sound analysis of the 8th edition of the staging system for NSCLC in a European cohort with clear-cut results.
I only suggest a shortening of the discussion.

Reviewer 2: anonymous

Date received: 11-May-2020
Reviewer recommendation: **Return to author for minor modifications**
Reviewer overall scoring: **Excellent**

Assessment Form scores: 5 = High/Yes; 3 = Medium/Adequate; 1 = Low

Is the subject area appropriate for the journal					
Does the title clearly reflect the paper's content?					
Does the abstract clearly reflect the paper's content					
Do the keywords clearly reflect the paper's content?					
Does the introduction present the problem clearly?					
Are the results/ conclusions justified?					
How comprehensive and up-to-date is the subject matter presented?					
How adequate is the data presentation?					
Are units and terminology used correctly?					
Is the number of cases adequate?					
Are the experimental methods/ clinical studies adequate?					
Is the length appropriate in relation to the content?					
Does the reader get new insights from the article?					
Please rate the practical significance.					
Please rate the accuracy of methods.					
Please rate the statistical evaluation and quality control.					
Please rate the appropriateness of the figures and tables.					
Please rate the appropriateness of the references.					
Please evaluate the writing style and use of language.					
Please judge the overall scientific quality of the manuscript.					
Are the methods used worthy of reproduction in greater deal?	No				

Comments to author: In the IASLC database with about 80,000 patients early stage patients preferably of Asian populations were considered as a basis for the changes of the 8th edition of the UICC and AJCC TNM classification of lung tumours. In general for all changes in new editions of TNM classifications, validation studies are necessary and would especially be helpful if they focused on those populations with lesser case numbers as a basis of the changes, in lung cancer therefore European populations.
The object of this study considering a large number of German patients 8final analysis: 1013) was to determine whether the 8th edition was more accurate in predicting long-term survival in a European population of surgically treated NSCLC patients. This is a well conducted and performed study with interesting and plausible results as to general survival dates. The authors found significant survival differences between the newly defined stages 1A1, 1A2 and 1A3 (previously 1A). We also found that the 8th edition of TMN classification was a significantly better predictor of long-term survival, compared to the 7th edition.
It would be helpful for the readers of this important paper to include the T, N and M descriptors in the stage designation, e. g. Stage IA (T1mi, Ta N0M0) etc.

Authors' Response to Reviewer Comments

Date received: 17-May-2020

Response to reviewer 1

# Reviewer 1 suggested that we shorten the discussion. This we have done (page 4,5).

Response to reviewer 2

# Reviewer 2 suggested that we include the T, N and M descriptors along with the tumor stages. We believe this is a good suggestion for improving the readability of the paper and have incorporated these explanations into the first table where the stages are listed and also in the text (page 2,4).

Comments by the Editor-in-Chief on Revised Submission

All reviewer concerns raised were addressed satisfactorily. The manuscript may be published in the present stage.
